# *Candida auris* Infection, a Rapidly Emerging Threat in the Neonatal Intensive Care Units: A Systematic Review

**DOI:** 10.3390/jcm13061586

**Published:** 2024-03-10

**Authors:** Rozeta Sokou, Alexia Eleftheria Palioura, Paschalia Kopanou Taliaka, Aikaterini Konstantinidi, Andreas G. Tsantes, Daniele Piovani, Konstantina A. Tsante, Eleni A. Gounari, Zoi Iliodromiti, Theodora Boutsikou, Argirios E. Tsantes, Stefanos Bonovas, Nicoletta Iacovidou

**Affiliations:** 1 Neonatal Intensive Care Unit, “Agios Panteleimon” General Hospital of Nikea, 18454 Piraeus, Greece; al.palioura@gmail.com (A.E.P.); paschaliakt@gmail.com (P.K.T.); kmaronia@gmail.com (A.K.); 2 Neonatal Department, National and Kapodistrian University of Athens, Aretaieio Hospital, 11528 Athens, Greece; ziliodromiti@yahoo.gr (Z.I.); theobtsk@gmail.com (T.B.); niciac58@gmail.com (N.I.); 3 Microbiology Department, “Saint Savvas” Oncology Hospital, 11522 Athens, Greece; andreas.tsantes@yahoo.com; 4 Department of Biomedical Sciences, Humanitas University, Pieve Emanuele, 20090 Milan, Italy; dpiovani@hotmail.com; 5 IRCCS Humanitas Research Hospital, Rozzano, 20089 Milan, Italy; 6 Laboratory of Haematology and Blood Bank Unit, “Attiko” Hospital, School of Medicine, National and Kapodistrian University of Athens, 12462 Athens, Greece; ktsante@yahoo.com (K.A.T.); atsantes@yahoo.com (A.E.T.); 7 East Sussex Hospitals NHS Trust, Hastings TN37 7PT, UK; elenigounari@gmail.com

**Keywords:** *Candida auris*, neonates, candida infection

## Abstract

(1) **Background:** In recent years, a global epidemiological shift in candidemia has been observed, marked by the emergence of resistant non-albicans Candida species. *Candida auris*, in particular, has become a significant global concern, causing infections in both pediatric and adult populations within healthcare settings. Despite its widespread impact, there is a limited understanding of the clinical course and transmission dynamics of neonatal systemic Candida auris infections, hindering effective prevention and management. This study focused on the epidemiologic data, the clinical presentation, risk factors, and outcome of *C. auris* infection in neonatal population. (2) **Methods**: A systematic review of the literature using PubMed and Scopus databases until December 2023 was conducted. (3) **Results**: A total of 24 relevant studies were identified, encompassing 476 documented cases of *Candida auris* infection in neonates. Prematurity emerged as a primary risk factor, alongside total parenteral nutrition, central line insertion, mechanical ventilation, and prior broad-spectrum antibiotic use. The mortality rate reached approximately 42%, with therapeutic details sparingly reported in 12% of cases. Treatment strategies varied, with amphotericin B predominantly used as monotherapy, while combination antifungal agents were used in 44% of cases. Notably, 97.4% of cases exhibited fluconazole resistance, and 67.1% showed resistance to amphotericin B. Limited data were available on resistance to other antifungal agents. (4) **Conclusions**: Despite the rarity of neonatal *Candida auris* infections, their global occurrence necessitates comprehensive preparedness in patient care. A deeper understanding of *Candida auris* pathogenesis is crucial for developing effective strategies to control and prevent neonatal infections caused by this pathogen.

## 1. Introduction

*Candida (C.) auris* is a fungal pathogen, considered to be an emerging global health threat due to its rapid spread across the world and the emergence of strains resistant to multiple antifungal agents [1,2]. It is associated with hospital-acquired and endemic nosocomial infections [2,3]. There are two ways in which *C. auris* can affect the human body. It can either reside on some areas of the body, such as the skin, rectum, or mouth, in a condition known as asymptomatic colonization. In this situation, a person remains asymptomatic but can still transmit the fungi to others. It can also cause serious infections by entering the bloodstream or open wounds. Invasive *C. auris* infection is the most common clinical manifestation in the Intensive Care Unit (ICU). It could cause sepsis, meningitis, pneumonia, myocarditis, and urinary tract infections with high mortality rates [4]. A recent meta-analysis by Chen et al. [5] revealed that mortality due to fungemia from *C. auris* was 39–45%, reaching up to 80% in patients with serious underlying health problems [6]. The main risk factors for invasive candidiasis include prolonged hospitalization in the ICU, the use of medical devices such as central venous catheters, medical comorbidities, long-term antibiotic therapy, and individuals of extreme ages, such as elderly adults and newborns [7]. The fact that *C. auris* has been referred to as “an often resistant and sometimes deadly fungal infection”, “a superbug and extremely drug-resistant infection”, and “*C. auris* sickens dozens”, makes it clear that there is something perilous about this recently recognized strain. *Candida auris* is a species of fungus that grows as yeast, isolated for the first time in 2009 in Japan from a patient’s ear, thus the name auris which means ear in Latin [8]. It was also recognized as a pathogen in 15 patients in Korea with chronic otitis media, 3 of whom were children [9]. Its ability to cause severe infections was first described in 2011 when the fungus was isolated from blood samples of patients with fungemia in South Korea [10]. However, according to recent studies, *C. auris* has likely been present since 1996, but it went unnoticed due to the difficulty of its identification. Cases of fungal infections were reported, but scientists were not aware that they were caused by this specific strain of fungus. The first case to date was retrospectively recognized through DNA sequencing of the isolation from the blood culture of a Korean pediatric patient that had undergone surgery in 1996 [10]. Since then, *C. auris* strains have been isolated globally in sporadic infections, hospital epidemics, or as colonization among patients, especially in the ICU. These isolations are increasing with geometric progression, at least in the USA.

Currently, the real impact and severity of *C. auris* infections are yet to be clarified due to their difficulty in being isolated with conventional techniques used in most laboratories [11]. In these clinical situations, it is necessary to perform additional tests such as molecular analyses to confirm the possibility of infection by *C. auris*. *Candida auris* possesses three characteristic drawbacks. Firstly, as mentioned previously, it is very difficult to be identified. Secondly, it is progressively becoming resistant to antifungal medication. Thirdly, it can easily spread within healthcare facilities and is very difficult to extinguish from surfaces, rendering infection control policies crucial. Therefore, on March 20th 2023, the CDC published an urgent warning, describing *Candida auris* as a global health threat, that has already spread to half of the states in the USA. Since 2019, the World Health Organization has considered it to be “an antimicrobial resistance threat”, and it is included in the “Antibiotic Resistance Threats in the United States” list [12,13].

During the coronavirus (SARS-CoV-2) pandemic, several epidemic outbreaks of invasive *C. auris* infections were reported in hospitalized COVID-19-positive patients in the United States, Brazil, India, Mexico and Pakistan. This demonstrates the rapid spread of *C. auris* despite strict hygiene protocols applied in the management of SARS-CoV-2. These reports note that a high number of severely ill patients with COVID-19 develop candidemia with high mortality [14,15,16,17,18]. Its ability to disperse from one patient to another in hospital grounds and also through medical devices, including thermometers, is unprecedented in fungi and causes great concern. Therefore, it is mandatory to apply strict strategic measures, in order to avoid the grave combination that enables transmissions in these emerging contagious threats. Recently, cases of *C. auris* candidemia have been reported in pediatric patients with COVID-19 [16,19]. The high incidence of *C. auris* candidemia as well as other multi-drug-resistant bacteremia reported during the COVID-19 pandemic could also be attributed to the reckless use of wide-spectrum antibiotics and prolonged hospitalization [20].

Candida infection is the third most common cause of late-onset sepsis in the Neonatal Intensive Care Unit (NICU). Invasive candidiasis is the most common invasive fungal infection in pediatric patients, with high mortality and hospital costs. *Candida albicans* and *Candida parapsilosis* are the most frequently isolated types of fungi in neonatal systemic candidiasis [21,22]; however, over the last few years, there has been a rise in cases of systematic *C. auris* infection reported in neonatal patients in NICUs worldwide. These outbreaks have a poor prognosis, despite antifungal therapy, as *C. auris* infection is often lethal in neonates [23,24,25]. The incidence of invasive candidiasis cases in NICUs has risen during the last few years due to advances in healthcare. Many factors are involved in this rise, including invasive procedures, such as catheterizations of central veins and arteries, gastrointestinal tract invasive procedures, and the use of broad-spectrum antimicrobial therapies in the context of neonatal sepsis [23]. These infections also complicate the clinical course of term neonates with underlying health problems that are hospitalized in the NICU [26]. There are various international guidelines on the management, therapy, and prevention of invasive candidiasis in neonatal patients, most of which are based on what is known about neonatal *C. albicans* and *C. parapsilosis* infections [27,28,29]. However, immune responses, clinical course, and the outcome of invasive candidiasis, in combination with antifungal prescription guidelines vary among Candida species [19,30,31]. Furthermore, there is limited information about the clinical course of systemic *C. auris* infection and its spread, which undermines the sufficient prevention and management of fungemia in neonatal patients [32,33]. Hence, there is a clinical need to understand the pathogenesis of *C. auris* infection during the neonatal period as well as to characterize the interactions between neonates and *C. auris*. This is the reason we conducted a systematic review in order to collect and update our knowledge and practices concerning the risk factors, clinical presentation, and outcomes of *Candida auris* infection in neonates.

## 2. Materials and Methods

We followed the methodology of a systematic review to identify, evaluate, and interpret the available research studies that addressed our research objective. A protocol was prepared, following the Preferred Reported Items for Systematic Reviews and Meta-analysis (PRISMA, presented as a Supplementary Material) guidelines [34], which is registered in the PROSPERO database (CRD42023417031) and used in this systematic review.

### 2.1. Inclusion Criteria

- Randomized controlled trials (RCTs), observational studies, case reports and case series;
- Population: neonates;
- Study design: studies focused on *Candida auris* infection, defined as positive blood, urine, CSF, or other sterile anatomical site culture in neonates.

### 2.2. Exclusion Criteria

- Surveys that included children and adults in the same group with neonates and did not offer information that solely involved the neonatal population;
- Review articles, systematic reviews and meta-analyses, and conference proceedings were excluded;
- Studies not published in the English language.

### 2.3. Research Objective

To obtain information on the clinical presentation, risk factors, and outcomes of *Candida auris* infection in neonates.

### 2.4. Main Outcome(s)

1. Clinical presentation of *Candida auris* infection in neonates;
2. Risk factors/characteristics of the affected population;
3. Outcomes of the infection including mortality.

### 2.5. Search Strategy–Data Source

This systematic review was performed from May 2023 to December 2023. We used the PubMed and Scopus databases, with the research end date set at 31 December 2023.

The used combination of key words: “*candida auris*”, “neonate”, “newborn”, “premature”, and “preterm” with logical operators Boolean (AND, OR). Moreover, in order to cover the full expansion of the available literature and reduce the risk of losing studies, we manually searched and reviewed the references of each chosen study, individually, as well as the references of previous systematic reviews on the topic.

### 2.6. Resolving Conflicts

The data extraction and quality assessment were conducted independently by two researchers (P.K.T., A.E.P.), with conflicts being resolved through discussion and consensus between the researchers or, if needed, with the involvement of a third researcher (R.S.).

### 2.7. Data Synthesis and Presentation

We recorded the data on a table according to the day of life that the infection occurred, existing comorbidities, risk factors, prematurity, administration of total parenteral nutrition, placement of central catheters, mechanical ventilation and previous use of broad-spectrum antibiotics, number of participants, number of events, and other relevant criteria for the classification of the study population (subpopulations of neonates include preterm infants, very low birth weight infants, those undergoing cardiac or other surgical procedures, infants with congenital malformations, and so on) research study design, date of publication, regardless whether there was chronological ambiguity or not, number of participants, and other relative criteria in order to collect and, if possible, analyze the results of the study. We described potential gaps in the data and offered suggestions for future investigation.

## 3. Results

**Figure 1:**
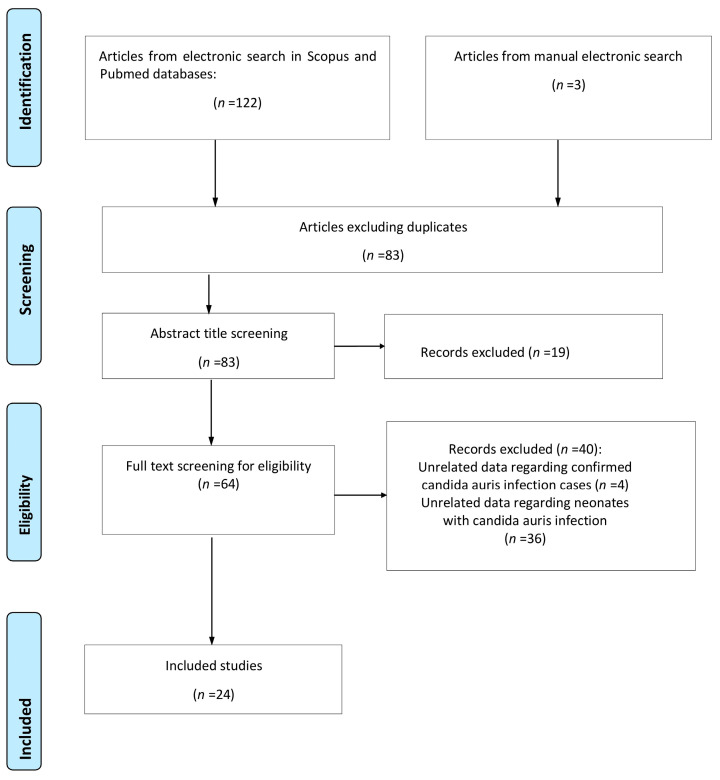
Flowchart of the systematic review.

A total of 125 studies were retrieved from the research of the electronic literature databases; 42 were duplicate entries and were removed. The removal of duplicate studies was achieved with the help of a management tool, which is used to remove duplicate studies, installed in the digital literature reference program (EndNote X8). After meticulously studying the titles and abstracts of the remaining studies, 19 of them were excluded either due to the fact that their subject did not serve the purpose of the study or because they met some of the exclusion criteria that was already evident from their title and abstract, while 12 reviews concerned the same field of inquiry but were not focused on the neonatal population. A careful study of the entire text of the remaining 64 studies revealed that only 24 of them met all the inclusion criteria and were therefore included in this systematic review. The flowchart is presented in Figure 1.

Of all the studies included in the present review, three concerned public health surveillance programs, four were laboratory-based surveillance series, five were case series, three were cross-sectional studies, and the remaining nine were cohort studies. The characteristics of each study, concerning cases of neonatal *C. auris* infection, are extensively presented in Table 1.

**Figure 2:**
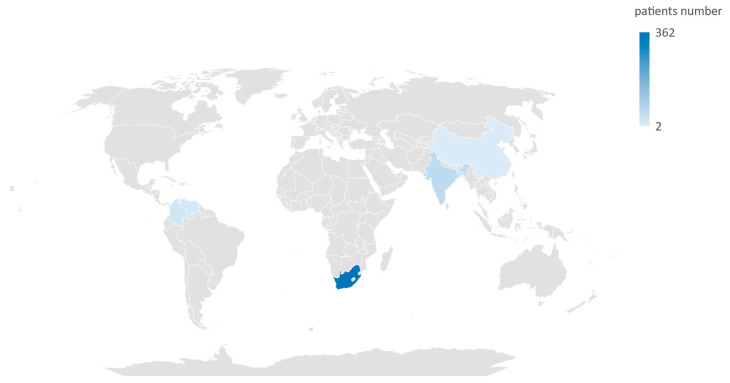
Map of countries where cases of *Candida auris* infection were documented in neonatal population.

In total, these studies assessed data from 476 neonates. The countries from which the cases of neonatal *C. auris* infection were documented are Bangladesh, China, Colombia, India, South Africa, and Venezuela (Figure 2).

The demographic data of the study population are presented in Table 2. Unfortunately, among the retrieved studies, there were lots of missing data. For the majority of cases, there were no data concerning the age of gestation. Of the 31 neonates with a documented gestational age, 20 were preterm neonates (64.5%), with a median gestational age of 28 weeks (IQR 26–34). The median patient age (among the 264 cases with reported data) during disease onset was 21 (range: 1–71) days.

Apart from prematurity, many other predisposing factors were documented, such as the administration of total parenteral nutrition (TPN), the placement of central line catheters, mechanical ventilation, and previous administration of broad-spectrum antibiotics (with piperacillin tazobactam and meropenem combined with vancomycin being the most used). As far as the underlying conditions are concerned, information was provided only for 42 neonates (8.8% of cases); congenital heart disease (14/42, 33.3%), congenital gastrointestinal disorders (10/42 neonates, 23.8%), and necrotizing enterocolitis (NEC, 4/42, 9.5%) were the most common comorbidities. From the available literature data, 20 out of the aforementioned neonates (48%) had a history of a previous abdominal or cardiac surgery. No neonate had previously received antifungal prophylaxis.

*Candida auris* was isolated from blood cultures (98.1%), urine (16.5%), peritoneal fluid specimen (2.4%), and cerebrospinal fluid (2.1%) (Table 3).

Information concerning the type of therapy was only provided for 57 cases. The choice of antifungal medication differed significantly between studies, with amphotericin B (AΜΒ) being the most frequently administered agent as monotherapy (Table 4). The mean duration of antifungal therapy (excluding neonates that passed away) was 27.85 days (range: 11–42 days). Information concerning the resistance of the pathogen to antifungal medication was only given for 79 cases. Specifically, resistance to fluconazole was mentioned in 77 out of the 79 cases (97.4%) and to AΜΒ in 53 cases (67.1%).

Data concerning the outcome of patients were provided for 93 cases, out of which the infection was successfully treated in 58.1% of patients, whereas 41.9% of neonates passed away. Information regarding survival was not available for 383 cases.

## 4. Discussion

Very little information is available for neonatal *C. auris* infection, while the potential severity of the disease requires timely recognition and appropriate management. To our knowledge, this is the first systematic review of the literature focused on the epidemiologic data, the clinical presentation, risk factors and outcome of *C. auris* infection in the neonatal population.

Invasive fungal infections (IFIs) are the main cause of morbidity and mortality in hospitalized neonates in the NICUs, especially preterm neonates and those with a very low birth weight (VLBW, <1500 g) [55]. These neonates are immunocompromised, exposed to broad-spectrum antibiotics, have an immature epithelial barrier and are subjected to invasive procedures, and consequently, they are at an increased risk for opportunistic fungal infections. The majority of IFIs are caused by Candida species [56]. Invasive Candidiasis (IC) is the third most common cause of late-onset sepsis in VLBW neonates and an important cause of morbidity and mortality in the NICU [56]. The frequency of IC in NICUs ranges between 0.5 and 20% and varies between centers and patient population and is inversely proportional to gestational age and birth weight, with a higher frequency documented in neonates with an extremely low birth weight (ELBW) [26,56,57]. However, cases of neonatal IFI caused by other fungal species are increasingly reported in the international literature. In recent years, we have witnessed a global change in candidemia epidemiology with the appearance of resistant, non-albicans species of Candida, with a particular emphasis on the increase in cases of *C. auris* infections. *Candida auris* is spreading globally and causing hospital infections in pediatric and adult populations, particularly in ICUs [58,59,60] and is included in the 2019 Emergency Threats Report of the Centers for Disease Control and Prevention (CDC) of the USA [12]. As is evident from the history of the discovery of this fungus, *C. auris* can cause severe ear infections. However, its main threat lies on its isolation from the bloodstream. Wound and soft tissue infections, osteomyelitis, central nervous system infections, and generalized infections in immunosuppressed patients have been described in the international literature [60]. According to our systematic review, we located 24 publications [23,24,25,32,35,36,37,38,39,40,41,42,43,44,45,46,47,48,49,50,51,52,53,54] from 2013 until today, in which 476 cases of *C. auris* infections are reported. Unfortunately, there was a lot of information missing among the retrieved studies. For the majority of the recorded cases, data regarding the age of gestation were not provided. However, it is noteworthy that, among the overall number of infants with reported gestational age, 64.5% were premature, suggesting that prematurity, as in other cases of IFI, is one of the main contributing factors to *C. auris* infection. Apart from prematurity, other contributing factors noted were the administration of total parenteral nutrition, the insertion of umbilical or other central lines, mechanical ventilation and prior administration of broad-spectrum antibiotics, which were also recognized in adult and pediatric patients with *C. auris* infection [60,61,62]. From the total number of patients with available data concerning underlying health problems, 33.3% had congenital heart disease, 23.8% had congenital gastrointestinal malformations, and 9.5% had necrotizing enterocolitis. These findings suggest that invasive infections caused by *C. auris* represent an emerging threat, not only for premature neonates but also for term neonates hospitalized in the NICU due to comorbidities, such as complex congenital heart disease or other congenital malformations requiring surgical correction [23,25]. Fungemia is a condition with high mortality, often as high as 50%, while the mortality rate for *C. auris* is reported to be between 40 and 60% [4,6]. The mortality rate we documented, in the total number of neonates with *C. auris* infection (93 cases), for which the studies provided information concerning the outcome of the infection, was quite high (approximately 42%), a finding consistent with the high mortality rate reported in studies focused on pediatric and adult patients [6,60].

*C. auris* has comparable characteristics with common candida species, concerning its force of infection and its ability to adhere to the host’s cell and form a biomembrane. However, in contrast to *C. albicans*, where the source of infection is usually the patient’s own intestinal microbiota, *C. auris* infections are mainly caused by the dispersion of the pathogen via the hands, from a colonized patient to another or from the colonized inanimate environment. *C. auris* causes systematic infections such as septicemia, soft tissue infections, and surgical site infections in seriously affected individuals of all ages, from premature infants to the elderly patients. It is also isolated from respiratory and urine samples, which it often colonizes [63]. A distinctive characteristic of *C. auris* is that it colonizes the patient’s skin and the hospital environment for extended periods of time and spreads from one patient to another within healthcare facilities, a characteristic not previously observed with other Candida species. These characteristics remind us of those seen in multi-resistant bacteria such as Acinetobacter or Klebsiella [63]. *C. auris* adheres to the skin due to a unique agent it possesses which does not exist in other Candida species called Surface Colonization Factor (Scf1) [64]. This agent provides the fungus with the ability to effectively adhere to the skin and central line catheters, resulting in infection. This discovery, recently published in the scientific journal *Science*, is significant because it might potentially lead to a new approach in treating this fungus through the development of new agents that inhibit the Scf1 factor and the fungus’s ability to adhere. Although reports of *C. auris* infections among neonates and children are rare, its appearance in different countries and continents worldwide represents a new reality that requires proper preparation across all aspects of patient’s healthcare. A better understanding of the mechanisms of pathogenesis will lead to strategies that aim to control and prevent *C. auris* infections in neonates. Information about experimental models of diffuse *C. auris* infection in adult mice have been reported. In these models, adult mice were intravenously infected with *C. auris*, revealing that the tissues with the highest fungal burden and invasion during the infection were the kidneys and the heart [65,66], as opposed to the experimental model of neonatal candidiasis conducted by Flores-Maldonado et al. [19], who realized that the liver and the brain of animal neonates were the tissues with the highest fungal burden and invasion during infection, indicating that the spread of the fungus during infection is different between neonates and adults. Therefore, there is great need to understand the pathogenesis of *C. auris* infection during the neonatal period and the characteristics of the interaction between *C. auris* and neonates.

Furthermore, these studies indicate that the pathogenesis and the outcome of systematic neonatal candidiasis varies between different strains of Candida. This is probably because different strains of Candida exhibit different patterns of virulence [67], antifungal sensitivity [68], and species-dependent recognition of the immune system [69]. Specifically, *C. auris* is a strain of fungus that causes different immune responses in comparison to other strains, such as *C. albicans* [70]. These differences in immune response are probably due to the composition of the cell wall of *C. auris*, where mannans and mannoproteins are the main inducers of the immune response, while in *C. albicans*, the main inducers of the immune response are glucans [71].

According to our systematic review, which includes 476 neonates with *C. auris* infection, the most common type of invasive infection was bloodstream infection (98.1%, 263/268 patients with available data on the site of infection). In our study, besides primary sepsis, other sites of infection were urinary tract infection (16.5%), meningitis (2.4%), peritonitis (2.4%), and skin abscess (1.2%). These findings are in accordance with information derived from studies on adult patients as well as with a recent systematic analysis conducted by Ashkenazi-Hoffnung and Rosenberg Danziger [60] which provides data on *C. auris* infection with a focus on children. According to the experimental model of Flores-Maldonado et al. [19], the spleen, kidneys, and lungs were the tissues of the mouse with the lowest colonization, while the liver showed the highest fungal invasion in the early stages of the infection and was associated with the presence of cellular infiltration. In contrast, the brain was the most susceptible tissue to invasion from *C. auris,* and the invasion persisted until the advanced stages of the infection, findings that were not confirmed by our systematic review. This could be primarily due to the high percentage of missing data (50.4%) in our review regarding the isolation site of *C. auris*. Additionally, we must take into consideration that in the experimental model, data concerning *C. auris* dissemination were evaluated via fungal burden and histopathological tissues analysis.

The clinical presentation of *C. auris* infections, in most cases, is nonspecific, and the providing a distinction from other types of systemic infections is often a challenge [72]. This fact is also reflected in our systematic review where, in the majority of neonates, *C. auris* infection had a similar clinical presentation with bacterial sepsis. As with other Candida infections, the diagnosis of invasive *C. auris* infection is made by isolating the yeast in blood cultures or other biological specimens from infected sites, clinically or radiographically ascertained. Identifying *C. auris* in cultures is often a prolonged procedure in comparison to more common types of Candida. The diagnosis of *C. auris* is extremely difficult as it is often confused with other rare fungi species (*C. haemulonii* ή *C. duobushaemulonii*, *C. famata*, *C. lusitaniae*, *Rhodotorula glutinis* ή Saccharomyces cerevisiae) using conventional fungal identification methods and demands the use of newer methods of identification, such as mass spectrometry MALDI-TOF MS, which are not widely available [60,73]. In order to overcome this problem, new precise and rapid diagnostic methods have been recently developed. Furthermore, morphological distinction detectors can be used for the rapid assessment of resistance mechanisms to echinocandins and azoles. *C. auris* has been included in the database of the VITEK-2 automated system since 2018. Despite being less available for routine testing, the molecular determination of sequence using polymerase chain reaction (PCR) analyses offers a definite identification of *C. auris*. Several molecular analyses have been developed, including conventional PCR assays, real-time PCR, magnetic resonance T2 sensing, and loop-mediated isothermal amplification (LAMP) assays [74,75]. As opposed to automated biochemical systems and MALDI-TOF MS, which depend on specimen cultures, DNA can be directly isolated from the patient’s samples, without the need for culture.

One of the main reasons of global concern about *C. auris* spreading is its sensitivity profile, which limits the choice of therapy. Most *C. auris* strains are resistant to fluconazole, cross-resistant to other azoles, and have variable increased minimum inhibitory concentrations (MICs) for amphotericin B [76,77]. Echinocandins have the lowest MIC for *C. auris* in comparison to other categories of systematic antifungal medication. However, resistance has been described even to this medication [78]. Data over time suggest that resistance rates to echinocandins are increasing [79]. Information derived from our systematic review, regarding resistance to antifungal medication, was only available for 79 cases. More specifically, 97.4% of the cases were resistant to fluconazole, and 67.1% of the cases were resistant to AMB. Data concerning the resistance to other categories of antifungal medication were limited. This finding raises concern over the management of neonatal *C. auris* infections as fluconazole and AMB are the most widely used antifungal medicine in NICUs. Moreover, fluconazole is, up to date, the first choice medication used as a prophylaxis against fungal infections in extremely low birth weight infants [80]. The treatment of *C. auris* presents with important challenges as it is resistant to various categories of antifungal medication, demanding changes in therapy due to treatment failure. Based on the information given above, the concern on resistance to azoles and AMB has led the CDC and Public Health England to recommend echinocandins as the first-line therapy against *C. auris* infections [75,81]. However, so far, no correlation has been established between in vitro sensitivity testing and clinical results. Observational data from hospital cases show high mortality rates between patients infected by *C. auris,* regardless of the choice of antifungal agent, both in adults [35,82] and children [32,44]. Additionally, past exposure to fluconazole and echinocandins has consistently been related to a high risk of *C. auris* infection [38,83]. In our review, data concerning the type of therapy were provided only for 12% of cases. The choice of antifungal medication differed significantly between studies, with AMB mostly used as monotherapy. The mean duration of antifungal treatment (excluding neonates that passed away) was 27.85 days (range 11–42). In 44% of cases, an invasive infection was treated with a combination of antifungal agents. Conclusions concerning differences in mortality rates according to antifungal treatment could not be drawn due to the small number of patients. In cases of *C. auris* pediatric infection, treatment recommendations are derived from the Infectious Diseases Society guidelines of 2016 [81] for the therapy of candidiasis, which suggest caspofungin or micafungin as both medications are approved by the FDA for use in children above 2 months of age. For neonates, amphotericin B deoxycholate is the first-line therapy choice. If sensitivity reports show increased (MIC) in all categories of antifungals, there is evidence suggesting that combination therapy with fluconazole and echinocandin, AMB, or azole effectively reduces MIC in vitro [84,85,86].

In settings with high rates of *C. auris*, some authors recommend antifungal prophylaxis with echinocandins in low birth weight premature neonates as an alternative to the typical prophylaxis with fluconazole [27]. This suggestion comes as a result of the high resistance that *C. auris* momentarily presents to fluconazole [32,87]. A small, comparative clinical study reported that the use of micafungin, in comparison to fluconazole, for prophylaxis against fungal infections in ELBW neonates was connected with lower rates of *C. albicans* infection [88]. Furthermore, the safety and pharmacokinetics of micafungin have been evaluated in VLBW [89,90]. As a result, within the context of an endemic–epidemic in a NICU, the prophylactic use of micafungin could be taken into consideration for high risk populations. However, as aforementioned, there is a concern that the echinocandins used could exert selective pressure, leaning in favor of the emergence of *C. auris*.

*C. auris* poses a serious challenge to the healthcare system due to its unique characteristics, including the extended transmission among patients, the persistence in hospital environments, misidentification via traditional laboratory methods, antifungal resistance, and the association with high mortality rates. The serious threat posed by *C. auris* has urged public healthcare services, throughout the world, to detect and report cases to health authorities.

Our study has certain limitations, and its findings should be interpreted with caution. Our main limitation was the high rate of missing data. The gaps and significant heterogeneity in recording basic information, such as the timing of disease manifestation, prenatal risk factors, demographic characteristics of neonates, mode of *C. auris* transmission, and diagnostic method, among the studies included in our review are evident. This fact served as a primary inhibitor to conducting a meta-analysis.

## 5. Conclusions

In conclusion, this study portrays that even though reports of neonatal *C. auris* infections are relatively rare, they have emerged in NICUs and seem to be replacing IFI caused by other fungal species. Their global emergence in many countries and across various continents represents a new reality that demands the effective planning of patient’s healthcare, including laboratory preparedness for adequate detection and high clinical awareness among healthcare providers for any unrecognized or rarely encountered species of Candida, in order to promptly apply infection control measures. Despite effective intervention and dramatic improvement in compliance with health protocols, the transmission of *C. auris* may persist even to a small extent. In this context, it is emphasized that prevention and early intervention are undoubtedly the preferred means to address this challenging pathogen. A future investigation focused on the risk stratification of patients as well as optimal choices of pre-symptomatic screening will offer important information in order to help healthcare facilities in preemptively identifying these outbreaks within the constraints of resources. The associations of *C. auris* with hospital outbreaks in the NICUs, invasive infections, high levels of resistance to antifungal medication, and high rates of mortality underline the importance of global collective efforts to raise awareness and limit its spread. Future research should address knowledge gaps concerning appropriate antifungal therapy.

## Figures and Tables

**Table 1:** Studies concerning cases of neonatal *C. auris* infection.

Authors	Country	Time Period	Type of Study	Number of Neonates	GA (Weeks)	Sex	BW (Grams)	Age at Diagnosis (Days)	Positive Blood Culture	Other Culture Site	Symptoms	Underlying Condition	Coinfection with	Treatment with	Survived	Died
Chowdhary [35], 2013	India	2009–2011	Case series	1	NR	F	NR	3	1	NR	NR	TEF, ICH, bacterial sepsis	Bacterial sepsis (not specified)	CASP		1
				1	NR	F	NR	10	1	NR	NR	ELBW, bacterial sepsis	Bacterial sepsis (not specified)	AMB	1	
				1	NR	F	NR	28	1	NR	NR	Pneumonia, late-onset sepsis	NR	AMB	1	
Calvo [36], 2016	Venezuela	03/2012–07/2013	Case series	1	NR	F	NR	30	1	NR	Sepsis	NR	NR	AMB, VOR	1	
				1	NR	F	NR	13	1	NR	Sepsis	colonic atresia	NR	AMB, VOR, CASP	1	
				1	NR	M	NR	17	1	NR	Sepsis	NR	NR	FLU		1
				1	NR	F	NR	23	1	NR	Septic shock	NR	NR	CASP	1	
				1	NR	M	NR	18	1	NR	Sepsis	NR	NR	FLU, VOR		1
				1	NR	M	NR	2	1	NR	NR	CHD and intestinal atresia	NR	CASP	1	
				1	NR	M	NR	12	1	NR	Sepsis	HIE, NEC	NR	VOR, CASP	1	
				1	NR	M	NR	11	1	NR	Sepsis	Surgical NEC	NR	AMB, CASP	1	
				1	NR	F	NR	18	1	NR	Sepsis	Congenital abdominal wall defect	NR	VOR	1	
				1	NR	F	NR	10	1	NR	Sepsis	HIE	NR	FLU	1	
				1	NR	M	NR	49	1	NR	Sepsis	RDS	NR	VOR, CASP	1	
Lockhart [37], 2017	Venezuela	2012–2015	Public health surveillance program	3	NR	NR	NR	NR	3	NR	NR	NR	NR	NR	NR	NR
Rudramurthy [38], 2017	India	2011–2012	Retrospective observational study	6	NR	NR	NR	NR	6	NR	NR	NR	NR	NR	NR	NR
Chen [39], 2018	China	2018	Case series	1	NR	M	940	71	1	NR	NR	NR	NR	FLU, then ITRA	1	
				1	NR	F	960	23	1	NR	NR	NR	NR	FLU, then ITRA	1	
van Schalkwyk [25], 2019	South Africa	2006–2017	Active national laboratory-based surveillance	20	NR	NR	NR	NR	20	NR	NR	NR	NR	NR	NR	NR
Dutta [40], 2019	Bangladesh	NR	Observational study	13	NR	NR	NR	NR	13	NR	NR	NR	NR	NR	NR	NR
Armstrong [24], 2019	Colombia	01/2015–09/2016	Outbreak investigation	5	36 (29–39)	NR	NR	NR	5	NR	NR	NR	NR	NR	4	1
Chakrabarti [41], 2020	India	04/2011–09/2012	Prospective observational study	6	NR	NR	NR	NR	6	NR	NR	NR	NR	NR	4	2
Berrio [42], 2020	Colombia	07/2014–10/2017	Retrospective microbiological review	7	NR	4M, 3F	NR	NR	7	NR	NR	NR	NR	3 AMB, 3 azoles, 3 caspofungin	3	4
Chandramati [32], 2020	India	2016–2017	Retrospective cohort study	1	31	M	1250	19 (+/−10)	1	NR	Skin abscess, fever, tachycardia	RDS	NR	VOR	1	
				1	28	F	900	19 (+/−10)	1	NR	Respiratory distress, lethargy	RDS, pneumothorax	NR	VOR		1
				1	28	M	850	19 (+/−10)	1	Urine	Respiratory distress, tachycardia	AREDF	NR	VOR, MIC	1	
				1	25	M	600	19 (+/−10)	1	Urine, lungs	Apnea, coagulopathy	RDS	NR	AMB, VOR	1	
				1	25	M	560	19 (+/−10)	1	Urine, CSF	Feeding intolerance	RDS, NEC	NR	VOR, MIC		1
				1	36	M	2460	19 (+/−10)	1	NR	Lethargy, abdominal distension	CHD	NR	AMB, VOR	1	
				1	37	M	2650	19 (+/−10)	1	Urine	Respiratory distress, tachycardia	Anemia, hepatosplenomegaly	NR	FLU, VOR		1
				1	31	M	1250	19 (+/−10)	1	NR	Apnea, lethargy, tachycardia	Inborn error of metabolism, renal failure	NR	FLU		1
				1	25	M	500	19 (+/−10)	1	NR	Apnea, lethargy	RDS, coagulopathy	NR	FLU		1
				1	40	M	2950	19 (+/−10)	1	Pus	Respiratory distress, tachycardia, skin abscess	MAS, BPD	NR	MIC, AMB	1	
				1	39	F	2750	19 (+/−10)	1	Urine, eye	Respiratory distress, tachycardia	CDH	NR	MIC, AMB	1	
				1	35	M	2520	19 (+/−10)	1	Urine	Respiratory distress, tachycardia	CDH	NR	MIC, AMB	1	
				1	31	F	1220	19 (+/−10)	1	Wound	Respiratory distress, tachycardia	Jejunal atresia	NR	MIC, AMB	1	
				1	35	M	2290	19 (+/−10)	1	NR	Respiratory distress	Jejunal atresia	NR	MIC, AMB	1	
				1	33	F	1430	19 (+/−10)	1	NR	Respiratory distress	TOF	NR	AMB, VOR	1	
				1	37	M	2670	19 (+/−10)	1	Urine	Fever, tachycardia	CDH	NR	VOR	1	
				1	36	F	2410	19 (+/−10)	1	NR	Fever, feeding intolerance	Jejunal atresia	NR	AMB, VOR	1	
Withers [43], 2021	South Africa	2019	Prospective observational study	14	NR	NR	NR	NR	14	NR	NR	NR	NR	NR	NR	NR
Ramya [44], 2021	India	08/2019–09/2020	Case series	1	26	F	675	7	1	NR	Long line induration	NR	NR	MIC	1	
				1	26	M	790	14	1	Budding yeast in urine NOT specified	Apnea	NR	NR	MIC		1
				1	25	M	620	9	1	NR	Apnea	NR	NR	MIC, VOR		1
				1	26	M	775	7	1	Budding yeast in urine NOT specified	Abdominal distension	NR	NR	MIC, VOR		1
				1	27	F	685	10	1	NR	Abdominal distension	NR	NR	MIC, VOR		1
Alvarado-Socarras [23], 2021	Colombia	2015–2017	Case series	1	35	NR	2300	12	1	peritoneal fluid	Fever	NEC	NR	CASP	1	
				1	39	NR	3100	14	0	Urine	Fever, thrombocytopenia	CHD	NR	FLU		1
				1	38	NR	2800	15	0	CHD, hydrocephalus, omphalocele		Kl.pneumoniae (wound) and A.Junii (blood)	14	CASP	1	
				1	39	NR	3200	37	1	Surgical sternal incision and pleural fluid	NR	CHD (hypoplastic left heart and anomalous venous return)	NR	FLU & CASP	1	
				1	38	NR	3400	14	1	0	NR	CHD (Ebstein anomaly)	NR	CASP & FLU	1	
				1	39	NR	3100	9	1	Urine (positive for C.tropicalis and R. ornitinolytica)	Unspecified respiratory symptoms	Dilated cardiomyopathy	NR	CASP		1
				1	38	NR	3300	16	0	urine	Thrombocytopenia	CHD (d-TGA)	NR		1	
				1	39	NR	3400	13	0	Central venous catheter tip	Thrombocytopenia	CHD and right hydronephrosis, NEC, Meckel’s diverticulum	NR	CASP	1	
Mishra [45], 2021	India	08/2020–09/2020	Cross-sectional study	1	27	NR	NR	NR	1	NR	Sepsis, refractory shock	NEC	NR	MIC, VOR	1	
Shuping [46], 2021	South Africa	2012–2017	Laboratory-based surveillance	7	NR	NR	NR	NR	7	NR	NR	NR	NR	NR	NR	NR
Chibabhai [47], 2022	South Africa	01/2016–12/2020	Retrospective laboratory analysis	14	NR	NR	NR	NR	14	NR	NR	NR	NR	NR	NR	NR
Mashau [48], 2022	South Africa	2014–2019	Cross-sectional study	60	NR	NR	NR	NR	59	1 (CSF)	NR	NR	NR	NR	NR	NR
Sathi [49], 2022	Bangladesh	2021	Cross-sectional study	1	NR	M	NR	15	1	NR	NR	NR	NR	VOR		1
				1	NR	M	NR	2	1	NR	NR	NR	NR	VOR		1
				1	NR	M	NR	12	1	NR	NR	NR	NR	VOR	1	
Cook [50], 2023	Various low- and middle-income countries	2018–2020	Prospective observational cohort study	18	NR	NR	NR	NR	18	NR	NR	NR	NR	NR	13	5
Sathi [51], 2023	Bangladesh	2021	Retrospective study	1	NR	F	LBW	6	1	NR	Septicaemia	NR	NR	NR	1	
				1	NR	M	LBW	15	1	NR	Septicaemia	NR	NR	NR		1
				1	NR	M	LBW	30	1	NR	Septicaemia	NR	NR	NR		1
				1	NR	M	NR	2	1	NR	Septicaemia	NR	NR	NR		1
				1	NR	M	LBW	1	1	NR	Septicaemia	NR	NR	NR		1
				1	NR	M	NR	22	1	NR	Septicaemia	NR	NR	NR		1
				1	NR	M	LBW	2	1	NR	Septicaemia	NR	NR	NR		1
Singhal [52], 2023	India	2015–2022	Retrospective observational study	6	>34	NR	NR	NR	6	NR	NR	NR	NR	NR	NR	NR
Shuping [53], 2023	South Africa	06/2019–06/2022	Point prevalence surveys	208	NR	102 F, 97 M	NR	21 days (IQR, 14–36)	NR	NR	NR	NR	NR	NR	NR	NR
Ramdin [54], 2023	South Africa	01/2013–12/2019	Retrospective cohort study	32	NR	NR	NR	NR	32	NR	NR	NR	NR	NR	NR	NR

Abbreviations: AMB, amphotericin B; AREDF, absent or reversed end-diastolic flow; BPD, bronchopulmonary dysplasia; BW, birth weight; CUSP, caspofungin; CDH, congenital heart defects; d-TGA, dextro-transposition of the great arteries; GA, gestational age; ICH, intracerebral hemorrhage; ITRA, itraconazole; ELBW, extremely low birth weight babies; F, female; FLU, fluconazole; HIE, hypoxic–ischemic encephalopathy; M, male; MAS, meconium aspiration syndrome; MIC, micafungin; NEC, necrotizing enterocolitis; NR, not reported; RDS, respiratory distress syndrome; TEF, tracheoesophageal fistula; TOF, tetralogy of Fallot; VOR, voriconazole.

**Table 2:** Demographic data of the study population.

		Reported Data	Missing Data
Gestational age (weeks)	Preterm	20/31 (64.5)	445/476 (93.5)
	Term	11/31 (35.5)	
Gender	Male	132/254 (52.0)	222/476 (46.6)
	Female	122/254 (48.0)	
Predisposing factors	Prematurity	55/71 (77.5)	405/476 (85.1)
	Total parenteral nutrition administration	25/94 (26.6)	382/476 (80.3)
	Central line catheter placement	41/94 (43.6)	382/476 (80.3)
	Mechanical ventilation	17/94 (18.1)	382/476 (80.3)
	Broad-spectrum antibiotic administration	18/94 (19.1)	382/476 (80.3)

Data are presented as absolute frequencies (percentages).

**Table 3:** The isolation site of *C. auris*.

Isolation Site	Reported Data	Missing Data
Blood specimen	263/268 (98.1)	208/476 (43.7)
Other isolation sites		
Urine	14/85 (16.5)	391/476 (82.1)
Peritoneal fluid specimen	2/85 (2.4)	
Cerebrospinal fluid specimen	2/85 (2.4)	
Central venous catheter tip	1/85 (1.2)	
Surgical sternal incision and pleural fluid	1/85 (1.2)	
Pus	1/85 (1.2)	
Wound	1/85 (1.2)	

Data are presented as absolute frequencies (percentages).

**Table 4:** Treatment strategies and survival rate.

Antifungal Therapy	Patients (n Reported Data/n all Cases, %)
Amphotericin B monotherapy	15/57 (26.3)
Amphotericin B and voriconazole	7/57 (12.3)
Amphotericin B and caspofungin	1/57 (1.8)
Fluconazole	5/57 (8.8)
Fluconazole and voriconazole	2/57 (3.5)
Fluconazole and caspofungin	2/57 (3.5)
Fluconazole and itraconazole	2/57 (3.5)
Micafungin	2/57 (3.5)
Micafungin and voriconazole	8/57 (14.0)
Micafungin and amphotericin B	2/57 (3.5)
Voriconazole	4/57 (7.0)
Voriconazole, amphotericin B, and caspofungin	1/57 (1.8)
Survived	54/93 (58.1)
Died	39/93 (41.9)

Data are presented as absolute frequencies (percentages).

## Data Availability

Data are contained within the article.

## Supplementary Materials

The following supporting information can be downloaded at: https://www.mdpi.com/article/10.3390/jcm13061586/s1, PRISMA checklist.
